# Image-Based Shrimp Aquaculture Monitoring

**DOI:** 10.3390/s25010248

**Published:** 2025-01-04

**Authors:** Beatriz Correia, Osvaldo Pacheco, Rui J. M. Rocha, Paulo L. Correia

**Affiliations:** 1 Instituto de Telecomunicações (IT), Instituto Superior Técnico, Universidade de Lisboa, 1049-001 Lisbon, Portugal; correiabeatriz2001@gmail.com; 2 Instituto de Engenharia Eletrónica e Informática (IEETA), Departamento de Eletrónica, Telecomunicações e Informática, Universidade de Aveiro, 3810-193 Aveiro, Portugal; orp@ua.pt; 3 RiaSearch Lda., 3870-168 Murtosa, Portugal; ruirocha@riasearch.pt; 4 Centro de Estudos do Ambiente e Mar (CESAM), Departamento de Biologia, Universidade de Aveiro, 3810-193 Aveiro, Portugal

**Keywords:** image-based shrimp monitoring system, aquaculture system, object detection and segmentation, Raspberry Pi, shrimp length estimation, shrimp width estimation, shrimp weight estimation, feed pellet attractiveness

## Abstract

Shrimp farming is a growing industry, and automating certain processes within aquaculture tanks is becoming increasingly important to improve efficiency. This paper proposes an image-based system designed to address four key tasks in an aquaculture tank with *Penaeus vannamei*: estimating shrimp length and weight, counting shrimps, and evaluating feed pellet food attractiveness. A setup was designed, including a camera connected to a Raspberry Pi computer, to capture high-quality images around a feeding plate during feeding moments. A dataset composed of 1140 images was captured over multiple days and different times of the day, under varying lightning conditions. This dataset has been used to train a segmentation model, which was employed to detect and filter shrimps in optimal positions for dimensions estimation. Promising results were achieved. For length estimation, the proposed method achieved a mean absolute percentage error (MAPE) of 1.56%, and width estimation resulted in a MAPE of 0.15%. These dimensions were then used to estimate the shrimp’s weight. Shrimp counting also yielded results with an average MAPE of 7.17%, ensuring a satisfactory estimation of the population in the field of view of the image sensor. The paper also proposes two approaches to evaluate pellet attractiveness, relying on a qualitative analysis due to the challenges of defining suitable quantitative metrics. The results were influenced by environmental conditions, highlighting the need for further investigation. The image capture and analysis prototype proposed in this paper provides a foundation for an adaptable system that can be scaled across multiple tanks, enabling efficient, automated monitoring. Additionally, it could also be adapted to monitor other species raised in similar aquaculture environments.

## 1. Introduction

Shrimp farming is a rapidly growing industry, driven by the rising global demand for seafood. By 2050, the fish and seafood demand is expected to increase by 50%, placing immense pressure on aquaculture farmers to improve efficiency while maintaining sustainability [1].

One of the key challenges in shrimp aquaculture is managing the high cost of feed, which accounts for 40% to 65% of total production costs [2]. Feed optimization is essential not only for economic reasons but also due to its environmental impact, as it contributes significantly to the industry’s carbon footprint [3]. Additionally, farmers must monitor for issues such as cannibalistic behavior, triggered by food scarcity or molting, which further highlights the need for continuous monitoring [4].

Currently, farmers rely heavily on manual observation to monitor feed consumption, growth, and population. This traditional approach is not only inefficient but also limits their ability to collect comprehensive data. Typically, shrimp growth is assessed by manually sampling a small portion of the population, a process that can be time-consuming and does not provide a comprehensive view of the entire tank. There is a clear need for automated systems that can continuously monitor shrimp, analyze pellet attractiveness, track shrimp growth, and accurately count the population. Such systems would greatly reduce labor requirements, improve the process of data collection, and enable farmers to make data-driven decisions for better management of shrimp tanks.

By automating the monitoring of shrimp and pellet attractiveness, the system proposed in this work provides a valuable tool for shrimp farmers, offering an efficient and scalable solution to address the challenges faced in modern shrimp farming. Tested at pilot scale under practical conditions at RiaSearch, a Portuguese research organization dedicated to aquaculture studies in nutrition, health, and sustainability, the objective of this work is to develop a prototype system capable of accurately estimating shrimp length and weight, counting the number of shrimps, and analyzing the attractiveness of pellets. The system focuses on extracting these key indicators from images captured during feeding periods, and its effectiveness is evaluated in a real aquaculture shrimp tank. Ultimately, this work serves as a foundation for future expansion, with the potential to adapt the system for simultaneous monitoring across multiple tanks, creating an integrated, automated network for shrimp farming management.

Recent advancements in automated systems and computer vision have started to transform traditional aquaculture practices, with a few works exploring their application in shrimp farming environments.

Nurmaida et al. [5] proposed a shrimp seed counting machine composed of three main parts: a seed container at the bottom, a section for cameras and sensors in the middle, and hardware components at the top. The system uses a Logitech C920e webcam to capture images of shrimp seeds, with data processed by a Raspberry Pi 4 board that powers the camera and an LCD screen for displaying results. Among the digital imaging approaches, Nurmaida et al. compared three CNN models for shrimp seed detection: Faster R-CNN ResNet50, Faster R-CNN ResNet101, and SSD MobileNetV2 [6,7,8,9], achieving the highest precision (80.1%) with Faster R-CNN ResNet101.

Huang et al. [10] developed an IoT-based system to monitor shrimp and analyze feeding behavior in turbid underwater environments often found in shrimp farms. The system aims to support future development of an automatic feeder by enabling real-time monitoring through the use of edge devices. To reduce server load and storage needs, image preprocessing and object detection are conducted locally on-site using a Raspberry Pi 3 board and an RK3399Pro Evaluation Board, from Rockchip, respectively. The processed data are then sent to a cloud server, which optimizes the system’s scalability. By distributing tasks between devices, parallel processing is achieved, resulting in faster system response times.

Nontarit et al. [11] developed an automatic feeding-tray lifting system designed to monitor and estimate shrimp growth. The system consists of a floating structure in a shrimp pond, equipped with a motor to raise a feeding tray out of the water. Images of the shrimp on the tray are captured by a Raspberry Pi Camera Module V2, processed by a Raspberry Pi Zero W, and then wirelessly transmitted to a remote server for analysis. For shrimp detection, Mask R-CNN ResNeXt [7,12] was employed, achieving a satisfactory F1 score of 73.31%, though challenges with overlapping objects were noted. Nontarit et al. also proposed two methods for estimating shrimp length: the “box measurement” method, which uses a bounding box around the shrimp but struggles with curved shrimp, and the more accurate “skeleton measurement” method, which applies skeletonization to the shrimp’s segmentation mask to measure length, effectively addressing curvature issues.

Hu et al. [13] proposed a deep learning-based system for monitoring white shrimp in turbid underwater environments. The system uses an underwater camera positioned over a green observation net to capture images of shrimp and bait. The detection model, YOLOv5, was applied both with and without image enhancement, with results showing that the enhancement did not significantly improve detection performance. For shrimp detection, this model obtained F1 scores of 97.40% and 98.10% with and without enhancement, respectively. The bait detection achieved a precision of 81.35% without enhancement. For further analysis of bait, Hu et al. employed a connected-component labeling method to estimate the area occupied by the bait. To estimate shrimp body length, the system uses a cropping method and calculates the hypotenuse of the bounding box as an estimate of length, applying a correction factor to improve accuracy by filtering out unrealistic length values.

Zhang et al. [14] developed a lightweight version of the YOLOv4 model [15] tailored for real-time shrimp counting. The system uses MobileNetv3 [16] as the backbone for feature extraction, optimizing it for processing speed. Additionally, it incorporates a feature pyramid based on SSP [17] and PANet [18] to enhance feature extraction, resulting in three effective feature layers. The lightweight model achieved strong performance with an F1 score of 83.15%, making it a suitable option for real-time applications in shrimp counting.

Thai et al. [19] implemented U-Net [20] for shrimp segmentation in aquaculture systems but faced challenges with touching or overlapping shrimps, where the model would often detect them as a single segment. To address this, they applied a marker-controlled watershed segmentation algorithm, which separates touching objects by generating markers based on Euclidean distance from the nearest background pixel and placing watershed lines to create clear boundaries. This method effectively improved the system’s ability to count shrimp and estimate lengths by providing more accurate segmentation. Additionally, they used the box measurement method to estimate shrimp length by enclosing the shrimp within the minimum bounding box, similarly to Nontarit [11].

Datasets of shrimp in aquaculture tanks are scarce, with the only annotated public dataset having been made available by Zhou et al. [21]. It features images of shrimp inside a bowl with clean water across various lighting and densities, used for instance segmentation with contrastive learning.

The present paper contributes to the development of image-based systems for shrimp aquaculture monitoring by presenting a comprehensive image-based prototype capable of extracting multiple indicators derived from aquaculture environments, specifically in clean water systems such as recirculating aquaculture systems (RAS). Addressing a gap in the industry, this work systematically details each development stage, from setup design to indicator extraction, providing a valuable framework for shrimp aquaculture. Additionally, the study contributes an annotated dataset tailored for multi-task applications, a resource that helps address the scarcity of publicly available data in this field. Moreover, it provides a comparative analysis of various length estimation techniques, applying them to the same image set for a robust side-by-side evaluation. Finally, it also explores methods for shrimp weight estimation and pellet attractiveness analysis, areas that remain underdeveloped in the existing literature, thus laying a foundation for advancements in these critical aspects of aquaculture monitoring.

## 2. Materials and Methods

This section details the development of the proposed prototype system, which utilizes a Raspberry Pi paired with a Raspberry Pi Camera Module 3 WIDE to capture activity within an aquaculture shrimp tank. It also addresses the complete process, from assembling the prototype setup to creating the ShrimpFarming dataset, along with the models and techniques applied for object detection and segmentation, as well as for the estimation of indicators such as shrimp dimensions and weight or pellet attractiveness.

### 2.1. Prototype Setup Configuration

The prototype was designed to meet a set of technical requirements to fulfill its functional objectives. First, it required a compact computer capable of connecting to an image sensor, capturing images, and managing storage. Internet connectivity was necessary to enable remote system management and troubleshooting, with potential for future server integration. Since the system relies heavily on visual data, a high-quality camera was essential to capture detailed, high-resolution images of the shrimp and pellets, with a wide enough field of vision to encompass the feeding area. To control image acquisition precisely, a manual trigger was implemented, allowing synchronization with feeding events. Given the high humidity levels and potential water splashes in the tank environment, the hardware needed protection to prevent malfunctions. Additionally, the camera setup had to mitigate light reflections from natural light entering through a large ceiling window, ensuring that image quality was not compromised. The prototype required a feeding plate that not only provided high contrast with the shrimp and pellets to facilitate detection but also featured a slight elevation to prevent pellet dispersal, ensuring that shrimp gather in a specific area for feeding. Lastly, scalability was a priority, ensuring that the system could be easily replicated across multiple tanks for broader application.

The development of the prototype was guided by these requirements and was oriented toward addressing the challenges inherent to the real-world scenario of the shrimp aquaculture tank.

The system’s core was built around a Raspberry Pi due to its ease of setup, flexibility, compact size, and efficient power usage. Its capabilities in managing image acquisition and data transmission make it ideal for scalable deployment in shrimp tanks. A Raspberry Pi Cam WIDE was selected for its wide field of view, capturing the entire feeding area in one frame. Both the Raspberry Pi and camera were enclosed in protective cases to safeguard against humidity and water splashes, with a built-in fan to prevent overheating. To control image capture, a JOY-IT COM-5WS joystick was used, allowing precise capture timing during feeding. This joystick provides flexibility for future system expansions, as its various positions can be repurposed for additional functionalities.

Several configurations for the camera positioning were tested. An acrylic enclosure was initially considered to reduce light reflections on the water’s surface, thereby enhancing image clarity.

**Figure 1:**
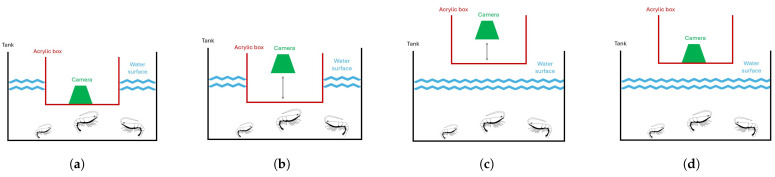
Setup configurations tested: (**a**) configuration 1; (**b**) configuration 2; (**c**) configuration 3; (**d**) configuration 4.

**Figure 2:**
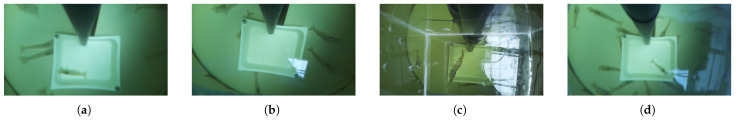
Images captured using the following: (**a**) configuration 1; (**b**) configuration 2; (**c**) configuration 3; (**d**) configuration 4.

However, after testing the four configurations, illustrated in Figure 1, unexpected challenges emerged. The captured images were consistently blurred, preventing the camera from properly focusing on the shrimp and pellets. This was due to small imperfections on the acrylic surface interfering with the camera’s ability to focus. Attempts to adjust the distance between the camera and the box improved clarity but reintroduced issues with water reflections, adding significant noise to the images, as illustrated in Figure 2.

**Figure 3:**
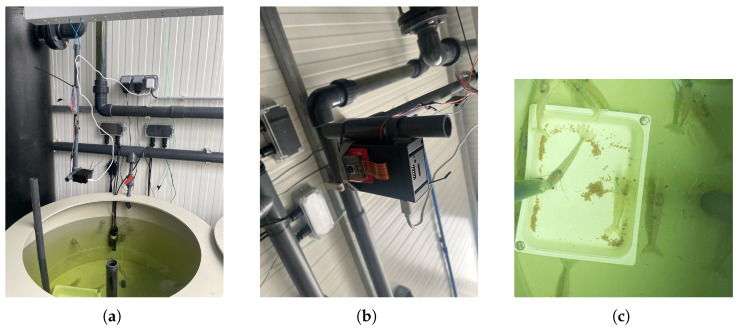
Final system setup: (**a**) setup overview; (**b**) Raspberry Pi+ Camera configuration; (**c**) example of captured image.

Given the challenges encountered, the idea of using an enclosure was discarded, and a more straightforward and effective solution was implemented. The camera is positioned 94.5 cm above the feeding plate, facing downward, capturing the feeding plate and the shrimp activity around it (see Figure 3a,b). The feeding plate, measuring 32 cm × 25.9 cm, is positioned in an area of the tank where light reflections are minimized. A joystick triggers image capture, ensuring control over the process of image acquisition. Figure 3c contains an example of a captured image using the setup configuration.

**Figure 4:**
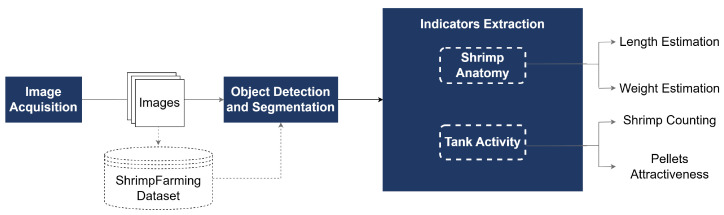
Proposed system architecture.

Regarding the system architecture (see Figure 4), the process begins with the image acquisition block, where images of shrimp feeding activity are captured. The images are then processed by the object detection and segmentation block, where models trained on the ShrimpFarming dataset proposed in this paper detect and segment key elements, such as shrimps, whereas a more traditional method is used for pellet detection. Then, the indicators extraction block analyzes the detected elements to derive key metrics for both shrimp anatomy and tank activity. This block can be divided into two groups: ‘Shrimp Anatomy’, focusing on shrimp length and weight estimation, and ‘Tank Activity’, which covers shrimp counting and monitoring pellet attractiveness.

With the architecture outlined, the following subsections present the proposed ShrimpFarming dataset and address each processing module of the system.

### 2.2. Shrimp Farming Dataset

The ShrimpFarming dataset includes a total of 1144 images, with a spatial resolution of 512 × 512 pixels. These images were captured over different days and times under natural illumination conditions. Additionally, two types of pellets, diets A and B, were also included to test the attractiveness of different types of food.

The training and validation sets were captured during three 20 min periods, with images taken every 4 s, covering two feeding periods and one non-feeding period. Of these images, 85% were used for training and 15% were used for validation. The test set consisted of four 20 min captures, following a structured protocol designed to emphasize early shrimp feeding moments, with images captured every 4 s for the first 2 min, every 8 s for the next 2 min, and then every minute for the remaining 16 min. This approach provided a more detailed view of shrimp behavior during the first few minutes after the pellets were inserted in the feeding plate.

In the dataset, feeding moments are labeled as “FeedX”, where X corresponds to the identifier of the specific capture. For example, Feed 3 and Feed 5 refer to two different captures, and each represents a unique set of images taken during those specific feeding moments.

The ShrimpFarming dataset includes two separate annotations per image, aligned with the extraction of indicators related to shrimp anatomy and tank activity. These annotations are stored in the YOLO format [22].

**Figure 5:**
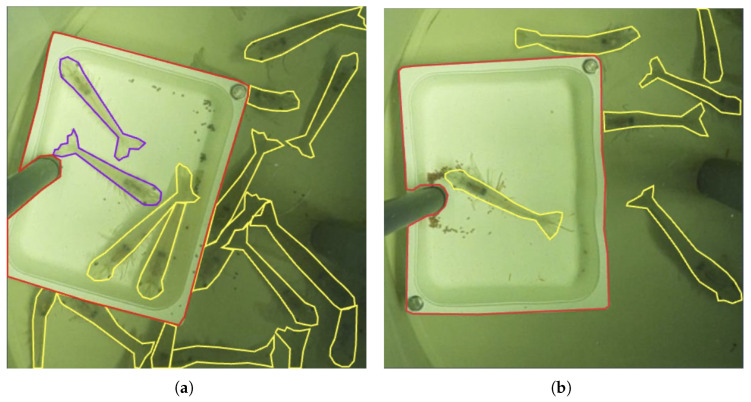
Manually annotated images using the two types of annotations described: (**a**) anatomy annotation; (**b**) activity annotation.

The anatomy annotation of the ShrimpFarming dataset was designed to indicate which shrimps are in suitable positions for accurate dimensions measurements. Segmenting the feeding plate is also essential, as its known dimensions provide a reference for shrimp size estimation. Three classes were defined (Figure 5a): the ‘plate’ class (red), representing the feeding plate; the ‘shrimp1’ class (yellow), for shrimps in suboptimal positions (e.g., curled or overlapped); and the ‘shrimp2’ class (purple), for shrimps in optimal positions, clearly visible and lying flat, thus suitable for precise measurement.

The activity annotation focuses on segmenting two key elements (Figure 5b): the ‘plate’ class (red), which represents the feeding plate as the central area of interest for monitoring pellet attractiveness, and the ‘shrimp’ class (yellow), which includes all shrimps within the tank, regardless of their body position.

The ShrimpFarming dataset will be made publicly available upon the publication of this manuscript to support further research and advancements in image-based aquaculture monitoring, addressing the current lack of datasets and annotated images in this field.

### 2.3. Object Detection and Segmentation

The YOLOv8m-seg model [23] was trained for both anatomy and activity annotations due to its robustness in handling complex shrimp segmentation tasks, which traditional methods, such as Canny Edge Detection, K-means Clustering, and Watershed Segmentation, struggled with. Preliminary tests using the Zhou et al. dataset [21] revealed that these traditional techniques required extensive fine-tuning and performed poorly for dense shrimp populations, due to overlapping, making them unsuitable for the real-world environment of an actual aquaculture shrimp tank. In contrast, YOLOv8 outperformed these techniques, demonstrating superior segmentation and capability of handling overlapping. YOLOv8 was selected over the newer YOLO models because it is more well-established and has more extensive community support, while still achieving state-of-the-art performance in computer vision tasks such as object detection and segmentation.

The YOLOv8m-seg variant was chosen for its optimal balance of model size, speed, and accuracy, delivering reliable segmentation with fewer parameters than larger models, yet with greater precision than smaller ones. After fine-tuning, the best-performing hyperparameters were found to be 50 epochs, a batch size of 16, and a learning rate of 0.01, enabling effective detection and segmentation across both the Shrimp Anatomy (anatomy annotations) and Tank Activity (activity annotations) group objectives. Two different models were trained: the Shrimp Anatomy model, which is used for the first group, and the Tank Activity model, which is used for the other group of objectives.

**Figure 6:**
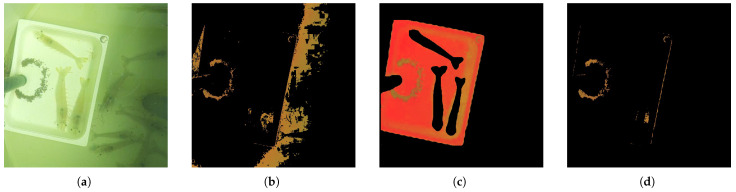
Pellet area estimation: (**a**) original image; (**b**) pixels in HSV range; (**c**) RoI for pellet detection; (**d**) pellets detected within RoI.

Pellet detection was performed by isolating specific HSV color ranges to identify pellets against the background. The HSV ranges used to detect the pellets were fine-tuned for each capture. The initial HSV segmentation successfully detected pellets in images from certain captures but struggled with variations in lighting, causing detection failures in other captures. Adjusting the considered threshold values may also lead to false detections (Figure 6b), including parts of shrimp or shadows within the tank.

Since the pellets are confined inside the plate area (RoI) and given that in this area of interest it is possible to remove the shrimp that were previously segmented, to refine the results, shrimp segmentations were subtracted from the image, and an RoI focused on the feeding plate (plate segmentation mask) was defined, reducing noise from the environment (Figure 6c,d).

### 2.4. Shrimp Length Estimation

**Figure 7:**
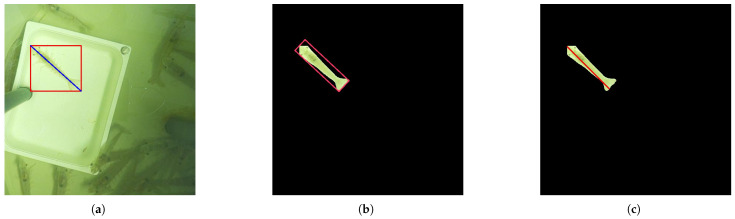
Shrimp length estimation example using the techniques: (**a**) Bounding Box Hypotenuse; (**b**) Minimum Bounding Box; (**c**) Probabilistic Hough Line Transform.

Three methods were considered for shrimp length estimation:

- The Bounding Box Hypotenuse method, applied by Hu et al. [13], calculates the shrimp’s length using the Pythagorean theorem, where the hypotenuse of the shrimp’s bounding box approximates its length (see Figure 7a). However, variations in shrimp orientation within the bounding box lead to inconsistencies in the calculated length, making this approach less accurate.
- The Minimum Bounding Box (MBBox) method offers a more precise measurement by generating a bounding box that tightly aligns with the shrimp’s orientation. The MBBox addresses issues related to shrimp alignment within the bounding box by tangentially touching both the shrimp’s head and tail, thus providing a more accurate length boundary, as shown in Figure 7b.
- The Probabilistic Hough Line Transform [24] is a more efficient variant of the traditional Hough Line Transform, designed to detect linear patterns within images. Unlike the standard approach, which processes all edge points to detect lines, the probabilistic version randomly selects subsets of points, significantly reducing computational load while maintaining effectiveness. This method was applied to the segmentation masks, successfully detecting lines that traverse from the rostrum to the tail of the shrimp. While it may not always align perfectly with the shrimp’s true length, it still provides consistent approximations. The detected lines often extend from one end of the shrimp’s body to the other, allowing for a relatively accurate estimation of length (see Figure 7c).

### 2.5. Shrimp Weight Estimation

**Figure 8:**
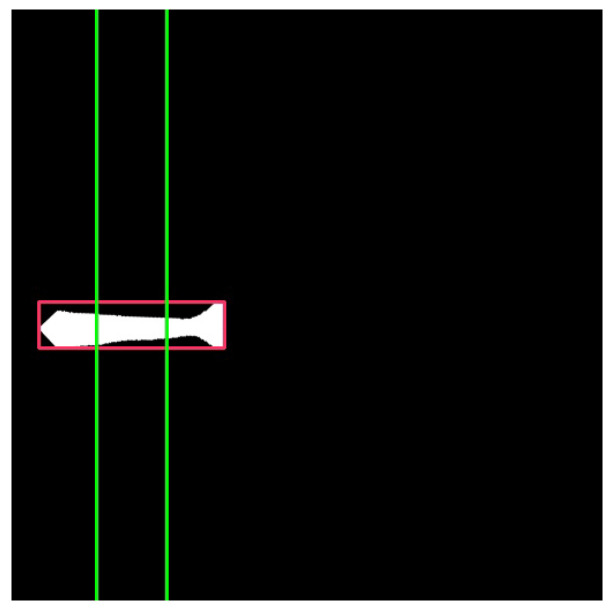
Shrimp width estimation: measurement considers the longest line drawn at 31% from both ends of its minimum bounding box.

The weight estimation method is based on the relationship between shrimp length, width, and weight, as recommended by the team exploring the aquaculture facilities. This approach starts by determining where to measure the shrimp’s width, which was found to align with a distinctive dark blob near the cephalon and thorax region, characteristic of *Penaeus vannamei*. Following the analysis of multiple shrimp images, it was determined that this marker is approximately 31% of the shrimp’s total length from the rostrum. The detected shrimp (‘shrimp2’ class) is rotated to align with the horizontal image axis, and two lines are drawn at 31% from both ends of its minimum bounding box, as illustrated in Figure 8. The line with the longer intersection with the shrimp segmentation mask is chosen as the shrimp’s width, as the head is typically wider than the tail.

Afterward, using the real dimensions of manually measured shrimps, a regression is created to correlate shrimp dimensions and weight.

### 2.6. Pellet Attractiveness

The study of pellet attractiveness is addressed here considering two different approaches: pellet distribution and shrimp activity on the feeding plate. Two types of pellets were tested—diet 1 (a standard shrimp feed containing fish meal, theoretically offering higher palatability) and diet 2 (a plant-based protein formulation with potentially lower palatability)—to observe differences in shrimp behavior, as only one is anticipated to align with their natural dietary preferences due to its superior attractiveness.

The pellet distribution approach tracks the decrease in area of the plate occupied by pellets over time using the HSV color space approach presented in Section 2.3 for pellet segmentation. As shrimp consume the feed, the occupied area is expected to diminish, allowing continuous measurement of the percentage of plate surface covered by pellets, providing an indirect gauge of the pellet consumption rate.

Regarding the second approach, monitoring shrimp activity around the feeding plate offers insights into pellet attractiveness, as shrimp gathering near the plate indicates interest in the food. A high concentration of shrimp suggests strong attraction, while fewer shrimp may signal reduced interest or less effective pellets. By analyzing the percentage of shrimp detected in the plate area over time, this approach tracks their interaction with the feed throughout the feeding period, providing a general view of shrimp response. To accurately monitor shrimp activity around the feeding plate, a threshold-based approach was applied to determine whether a shrimp was “inside” the plate area. Specifically, if at least 30% of a shrimp’s segmentation mask overlapped with the defined plate region, the shrimp was considered to be within the plate area. This method effectively filters out shrimp that may only partially intersect the plate boundary, focusing instead on those actively engaging with the feed or approaching the plate.

The same capture protocol used for the test set of the ShrimpFarming dataset is also used for the pellet attractiveness analysis; however, the captures last 30 min instead of 20 min.

### 2.7. Evaluation Metrics

To evaluate the accuracy of the proposed methods, the following metrics were used:

- Mean Absolute Percentage Error (MAPE): measures the average percentage difference between the predicted values (${\hat{y}}_{i}$) and the actual values ($y_{i}$). It is calculated as
  $$\mathrm{MAPE}=\frac{1}{n}\sum_{i=1}^{n}\left|\frac{y_{i}-{\hat{y}}_{i}}{y_{i}}\right|\times 100,$$
  where *n* is the number of observations. Lower MAPE values indicate higher accuracy.
- Mean Absolute Error (MAE): determines the average absolute magnitude of errors between predicted and actual values. It is defined as
  $$\mathrm{MAE}=\frac{1}{n}\sum_{i=1}^{n}\left|y_{i}-{\hat{y}}_{i}\right|.$$
- Root Mean Square Error (RMSE): a common metric that calculates the square root of the average squared differences between predicted and actual values. It is given by
  $$\mathrm{RMSE}=\sqrt{\frac{1}{n}\sum_{i=1}^{n}{\left(y_{i}-{\hat{y}}_{i}\right)}^{2}}.$$
  RMSE emphasizes larger errors due to the squaring process, making it more sensitive to outliers compared to MAE.

## 3. Results

This section presents results obtained in a real-world shrimp tank, using the Shrimp Anatomy model for size estimation methods, the Tank Activity model for shrimp counting and shrimp activity, and the HSV-based approach for pellet segmentation used for pellet attractiveness.

### 3.1. Object Detection and Segmentation

The Shrimp Anatomy model, trained with the anatomy annotations, performs well overall, with the ‘plate’ class achieving near-perfect precision, recall, and F1 scores in both bounding box detection and segmentation tasks (see Table 1), which is expected due to the plate’s distinctiveness in the images. The two shrimp classes, considered for facilitating the identification of shrimps in optimal conditions for measurements, score lower, as their similarity adds complexity to the detection task. The ‘shrimp2’ class, in particular, achieved lower performance metrics, likely due to the model’s difficulty in determining the shrimp’s optimal position, given the limited amount of data used for training. Its segmentation mask metrics are still relatively good with an F1 score of 0.80.

**Figure 9:**
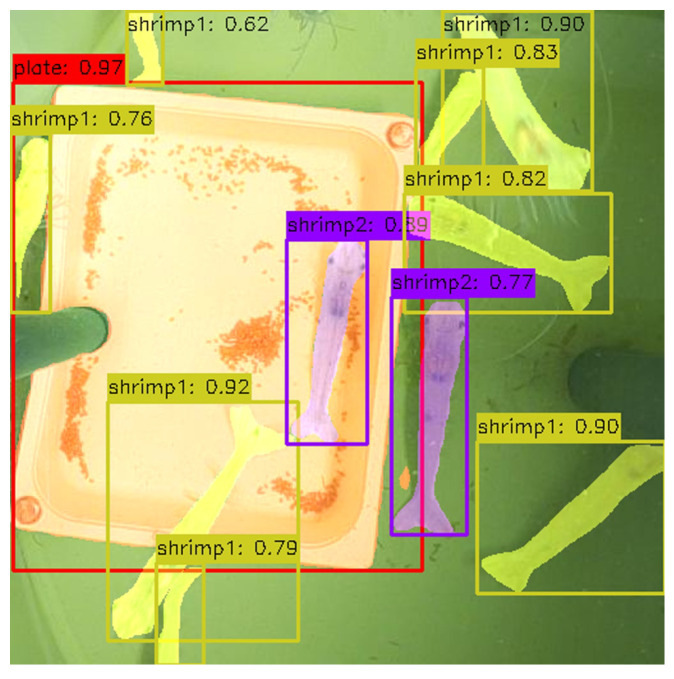
Example of a prediction using the Shrimp Anatomy model: ‘plate’ (red), ‘shrimp1’ (yellow), and ‘shrimp2’ (purple).

Figure 9a includes an example of a prediction using this model around the feeding plate. The image contains the detection and segmentation of the two shrimp classes and the feeding plate.

The Tank Activity model, trained using the activity annotations, shows strong performance in both detection and segmentation tasks (see Table 2). As expected, for the ‘shrimp’ class, the model’s performance improves compared to the Shrimp Anatomy model, as it deals with only a single class for shrimps. Precision (0.943) and recall (0.935) are crucial for the primary objective of shrimp counting, ensuring accurate detection.

**Figure 10:**
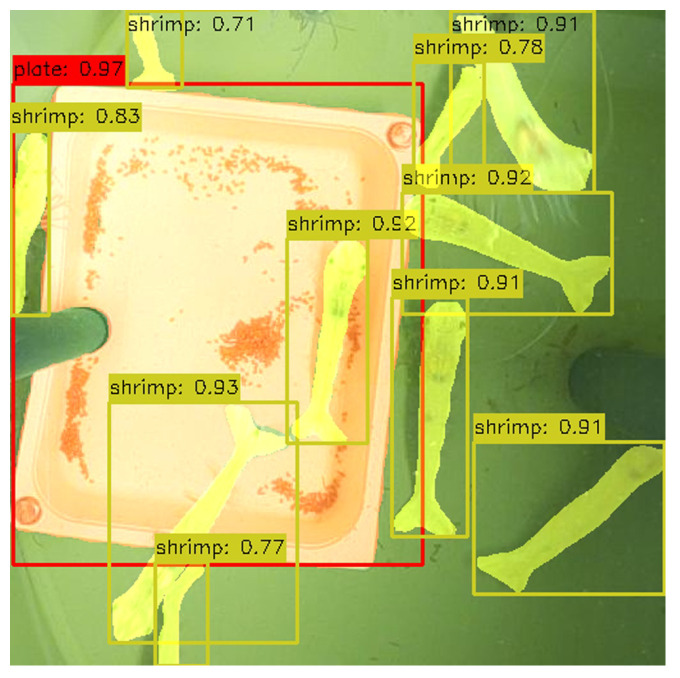
Example of a prediction using the Tank Activity model: ‘plate’ (red), ‘shrimp’ (yellow).

Figure 10 includes an example of a prediction using this model, illustrating shrimp detection and segmentation, as well as feeding plate detection.

Nontarit et al. [11] used a CNN with ResNeXt, reporting lower precision (0.745) and recall (0.722) compared to the YOLOv8 model used in this work. While Hu et al. [13] reported a precision and recall of 0.973 and 0.975, respectively, with YOLOv5, direct comparisons are difficult due to differences in datasets and conditions, though YOLO clearly outperforms the CNN model from Nontarit et al.

For the task regarding shrimp counting, the Tank Activity model will be used, since it obtains better detection results. The shrimps detected are then compared with the ground truth to evaluate the performance of detection and counting.

### 3.2. Shrimp Length Estimation

Shrimp length estimation methods were assessed using a ground truth based on manually measured shrimp lengths, calculated by selecting head and tail points for 60 individual shrimp and converting pixel distances to centimeters using the plate’s known dimensions. The ground truth of the corresponding shrimp is then compared with the length estimation obtained from the evaluated methods, which use the segmentation mask of class ‘shrimp2’ predicted by the Shrimp Anatomy model.

Looking at Table 3, which presents the error metrics calculated using the 60 manually measured shrimps, the MBBox method consistently performed best, achieving the lowest error rates, with a mean absolute percentage error (MAPE) of 1.56%, a mean absolute error (MAE) of 0.23 cm, and a root mean square error (RMSE) of 0.37cm, making it the most reliable method. The Hough Line method also delivered good results, with a MAPE of 2.95%, positioning it as a solid alternative. In contrast, the Hypotenuse method yielded higher errors (MAPE of 10.87%) due to its sensitivity to the shrimp’s alignment angle relative to the x and y axes.

Comparing these findings with existing studies, Nontarit et al. [11] and Thai et al. [19] also reported high accuracy with MBBox, reinforcing its reliability for shrimp length estimation. Although Hu et al. [13] referenced the Hypotenuse method, no specific results were provided. In conclusion, MBBox emerges as the most effective method, while Hough Line performs well in this context, as our algorithm previously selected shrimps that were in the ideal position for measurement (class ‘shrimp2’).

### 3.3. Shrimp Weight Estimation

The width estimation method described in Section 2.5 achieved promising results with a MAPE of 4.19%, a MAE of 0.15 cm, and a RMSE of 0.19 cm. Given the low error metrics for both length and width estimations, it is reasonable to expect that the weight estimation will also exhibit low error. Additionally, the polynomial regression method applied to these measurements also provides a strong fit to the data, further supporting the reliability of the weight estimates.

**Figure 11:**
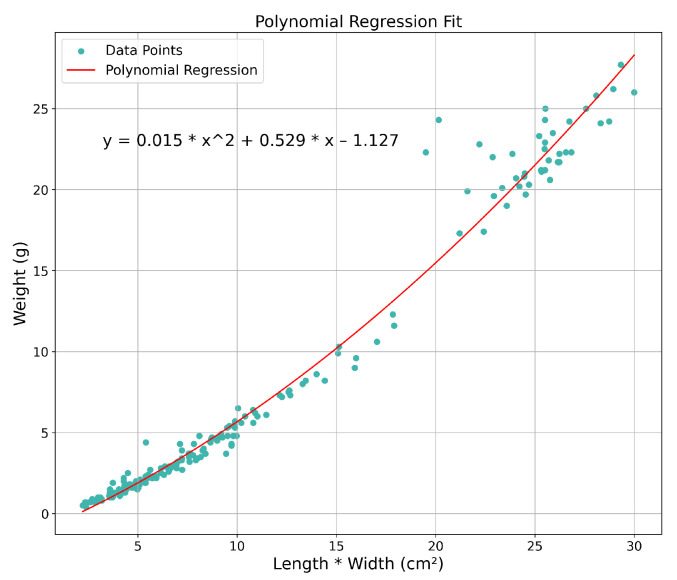
Polynomial regression to estimate weight.

Once the length and width are estimated, a regression model is used to predict the shrimp’s weight. Using data previously collected from 186 shrimps at RiaSearch, a polynomial relationship between these dimensions and weight was established, illustrated in Figure 11, providing a reliable model for accurately predicting shrimp weight from length and width measurements, supporting the overall monitoring system.

By estimating the shrimp’s dimensions and, consequently, its weight, this analysis offers valuable insights into monitoring growth rates during the early stages of shrimp development. Additionally, this approach can potentially be used to indirectly assess the nutritional quality of the pellets being used, as a slower growth rate might indicate that the feed is not providing the necessary nutrients to support optimal growth size, enabling adjustments to feeding strategies or pellet formulation.

### 3.4. Shrimp Counting

The shrimp detection of the Tank Activity model was used for shrimp counting as it detects shrimps without distinguishing between their positions, aligning well with the goal of simply counting shrimp rather than identifying specific characteristics.

The current camera setup captures only a portion of the aquaculture tank, limiting its field of vision to the shrimp within that area. To achieve comprehensive shrimp counting across the entire tank, the camera position would need to be readjusted to cover the full tank area.

The effectiveness of shrimp counting was evaluated by comparing predictions with ground truth annotations from four captures of the test set of the ShrimpFarming dataset (“feed3”, “feed4”, “feed5”, and “feed6”). A confidence threshold of 0.60 was used to balance detection accuracy and minimize false positives. Shrimp counting results are reported in Table 4 achieved an average MAE of 0.97, indicating that its shrimp count predictions are, on average, within one shrimp of the actual count, demonstrating reliable accuracy. The average RMSE of 1.38 further underscores the model’s consistency, with its closeness to the MAE suggesting stable performance without substantial outliers. The model’s MAPE across the four captures averaged 7.17%, though “feed4” exhibited a higher MAPE of 9.23%, which likely results from poor image quality caused by slight water turbidity or poor light conditions, which can hinder the detection of shrimps.

Thai et al. [19] also performed shrimp counting using a Marker-Controlled Watershed technique, achieving a MAE of 0.16. However, their work environment differed significantly from this work, with fewer overlapping shrimp in their images. In contrast, the environment in which the proposed prototype is inserted frequently encounters shrimp overlapping, complicating accurate counting. Additionally, some shrimps also occasionally hide beneath the feeding plate’s edge, further challenging detection and counting accuracy.

### 3.5. Pellet Attractiveness

Regarding the analysis of pellet attractiveness, given that pellet detection is not performed with a trained model and attractiveness is inherently challenging to quantify, evaluating the results poses certain challenges. As a result, the analysis focuses on qualitative insights rather than precise metrics.

**Figure 12:**
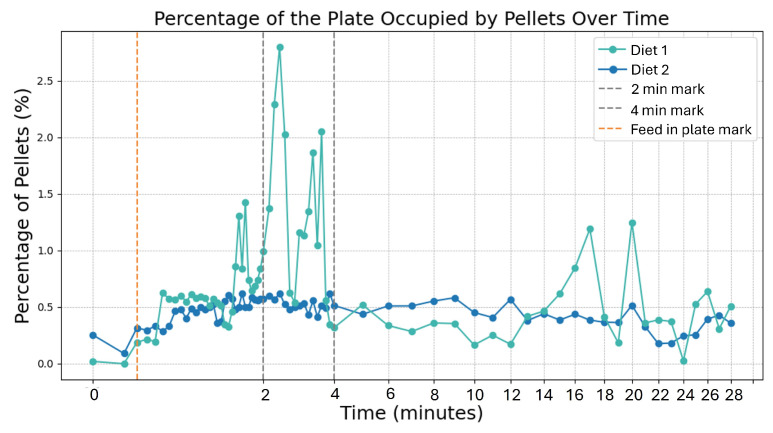
Pellet distribution during feeding periods using the following: diet 1 (green)—a standard shrimp feed containing fish meal, theoretically offering higher palatability, and diet 2 (blue)—a plant-based protein formulation with potentially lower palatability.

Figure 12 illustrates the plots for each type of pellet: diet 1 (green line) and diet 2 (blue line). These plots contain orange and grey vertical lines that serve as visual markers: the orange line marks when pellets were introduced, providing a reference for behavioral changes, while the grey lines indicate changes in image capture intervals—initially every 4 s, then 8 s, and finally, every minute. Both plots illustrate an initial increase in the area occupied by pellets on the plate shortly after they are introduced due to the pellets spreading across the plate’s surface. Throughout the capture period, there are shadows in the plate that fall within the considered HSV range due to lightning variations, hence the frequent oscillations and spikes, which is clearly visible for the green line, where the initial percentage is around 0.6% but subsequently spikes to 2.8%, as the pellets are spread over the plate surface due to water movements caused by the presence of shrimps over the feeding area. Then, the percentage values for the green line on average correspond to reality, and in minute 24 there are actually no more pellets in the plate; however, due to the limitations already mentioned, the percentage of area occupied then increases to values close to 0.5%. Regarding diet 2 (blue line), its percentage variations are less accentuated; however, the average value of area occupied by the pellets on the plate remains approximately the same, despite being eaten. Similarly to diet 1, in the last captures, there is a sudden increase in pellet area caused by segmentation errors, which causes overestimation. The percentage of pellets in minutes 22 and 23 approximately depict the real final percentage area of pellets. Although the plots suggest that diet 1 pellets occupy a slightly larger final area compared to diet 2 pellets, in reality, nearly all diet 1 pellets were consumed, while some diet 2 pellets remained uneaten.

**Figure 13:**
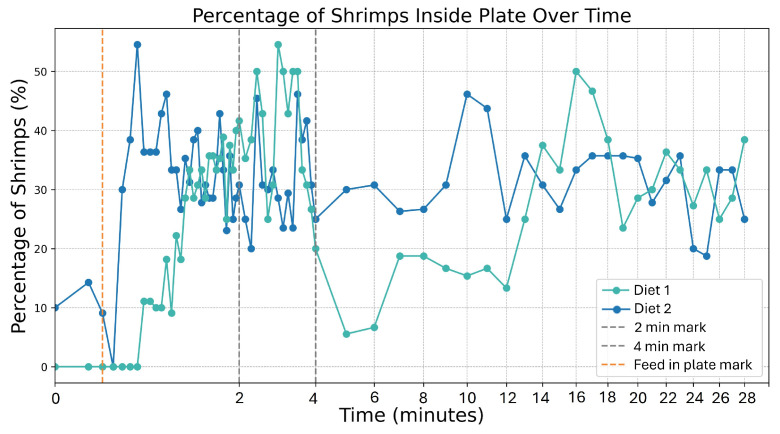
Shrimp activity inside plate during feeding periods using the following: diet 1 (green)—a standard shrimp feed containing fish meal, theoretically offering higher palatability, and diet 2 (blue)—a plant-based protein formulation with potentially lower palatability.

Shrimp activity was the second approach used to analyze pellet attractiveness. By looking at Figure 13, it is possible to see that the activity in the plate increases for both plot lines after the pellets are inserted, especially for diet 2 (blue line). For diet 1, there is a big decrease of approximately 40% around minute 5, which might suggest that the shrimps might have taken the pellets and left the plate area, which is a common behavior. Concerning diet 2, the capture presents very consistent shrimp activity around the plate; however, in the end, there were more diet 2 pellets left compared to the diet 1 pellets, which might indicate that the shrimps were not consuming the diet 2 pellets, even though more shrimps were swimming over the plate area.

Overall, the shrimp activity approach offers a more stable method for evaluating pellet attractiveness, as it is less influenced by external factors such as lighting variations, pellet overlap, and pellet stacking, all of which can introduce inconsistencies in the attractiveness analysis. However, a limitation of this approach is that shrimp detected within the plate area may not necessarily be consuming the pellets, meaning their presence alone might not always indicate effective pellet consumption. In contrast, monitoring pellet distribution provides a more direct measure of attractiveness by offering insight into the rate and quantity of pellets actually consumed. This method allows for a more precise assessment of pellet effectiveness based on the observed decrease in pellet coverage over time. However, since this method is more vulnerable to lightning conditions which impact the accuracy of pellet detection, it should be employed under diffuse artificial illumination conditions.

## 4. Conclusions

The results reported in this work demonstrate the strong performance of the YOLOv8 model for shrimp detection and segmentation, even under challenging underwater conditions, aligning with findings by Hu et al. (YOLOv5) and surpassing the performance of Nontarit et al.’s CNN model with ResNeXt [11]. However, the Shrimp Anatomy model struggled slightly with the ‘shrimp2’ class, likely due to its limited representation in the training set and the visual similarity between the shrimp classes. For pellet detection, the HSV color space method showed effectiveness under stable conditions but was prone to errors due to lightning variability, highlighting its limitations. Notice that in standard production conditions, shrimp aquaculture tanks typically use constant artificial illumination, which would mitigate the observed variability. In shrimp length estimation, the MBBox method achieved excellent results (MAPE of 1.56%), in line with the accuracy reported by Nontarit et al. [11] and Thai et al. [19], confirming MBBox as a reliable length estimation approach. It should be noted that water fluctuation can have a potential impact on shrimp length measurement if the images suffer distortions. Although the expected impact is small, a human screening of the images used for length estimation is advisable. The adopted shrimp weight estimation approach demonstrates a strong correlation between shrimp length, width, and weight, supporting its viability. Although it accumulates errors from both length and width measurements (MAPE of 4.19%), these errors are small, resulting in minimal overall error in weight estimation. This is of great value to shrimp farm management, as typically shrimp would need to be taken out of the tank for measurement, often leading to stress in the shrimp. In the envisaged use case, to evaluate the growth and weight gain of shrimp, the experts in shrimp feeding sample around 30% of the population and manually weigh and measure the size (with natural consequences for the animals’ well-being). The estimates obtained by the developed methods are good enough for use in this case. Shrimp counting achieved an average MAPE of 7.17%, demonstrating YOLOv8’s capability of detecting shrimps under these conditions; however, this reasonable result is directly affected by the conditions and the characteristics of the dataset. Therefore, a more robustly trained model will improve the shrimp counting results.

The pellet attractiveness analysis suggests that both approaches have their advantages and disadvantages, and that it is not possible to conclude which approach is better. However, in environments in which there are no light variations, the pellet monitoring approach seems to be the most promising because it can efficiently detect the pellet area, hence providing insight into the rate at which the feed is eaten.

## 5. Future Work

Although the existing prototype system has yielded encouraging outcomes in the detection and analysis of shrimp and pellet attractiveness, there is future work that could focus on improving the overall system’s performance and robustness.

One significant area of improvement is the setup. Placing the camera underwater in a waterproof housing could improve image quality, reduce reflection issues, and therefore improve the results. Other types of sensors could also be further explored: stereo camera, laser scanner, or hyperspectral camera.

As a further development of the overall setup, transmitting images to an external server for processing would offload tasks from the Raspberry Pi, enhancing resource efficiency and enabling future multi-tank scalability for comprehensive analysis of all the tanks.

Expanding the dataset with more images, particularly of ‘shrimp2’ class, would address class imbalances and improve the Shrimp Anatomy model’s accuracy. A larger dataset may eventually allow for the combined detection of both shrimp classes without having the need for two separate annotations.

Improving the ground truth measurements for length and weight using physical measurements rather than manual pixel selection would also help to better understand the accuracy of the estimations. For length estimation, keypoint detection (pose estimation) could also be an effective approach to be explored in the future.

Regarding pellet detection, a deep-learning model could replace the HSV method to achieve more reliable segmentation by performing semantic segmentation, as it would better adapt to lighting and environmental changes. Finally, a more extensive image capture campaign should be conducted to allow for better validation of the proposed methods.

As a summary, we believe that the work reported in this paper lays a solid foundation for automating traditionally manual tasks in aquaculture, although there is still room for improvement. Despite the limitations discussed, the methods presented streamline some aquaculture processes and provide comprehensive insights into shrimp tank dynamics. Overall, this work demonstrates the potential of an image-based system in shrimp aquaculture, paving the way for future advancements to enhance accuracy and robustness.

## Figures and Tables

**Table 1:** Evaluation metrics of the trained YOLOv8m-seg using anatomy annotation.

	Bounding Box					Segmentation Mask				
Classes	Precision	Recall	F1 Score	mAP50	mAP50-95	Precision	Recall	F1 Score	mAP50	mAP50-95
all	0.897	0.895	0.896	0.925	0.840	0.899	0.898	0.898	0.926	0.750
plate	0.997	1	0.998	0.995	0.995	0.997	1	0.998	0.995	0.982
shrimp1	0.880	0.897	0.888	0.937	0.757	0.886	0.904	0.895	0.942	0.626
shrimp2	0.814	0.788	0.801	0.842	0.766	0.814	0.789	0.801	0.842	0.642

**Table 2:** Evaluation metrics of the trained YOLOv8m-seg using activity annotation.

	Bounding Box					Segmentation Mask				
Classes	Precision	Recall	F1 Score	mAP50	mAP50-95	Precision	Recall	F1 Score	mAP50	mAP50-95
all	0.970	0.960	0.965	0.984	0.908	0.967	0.969	0.968	0.986	0.833
plate	0.996	0.985	0.990	0.991	0.991	0.996	0.985	0.990	0.991	0.987
shrimp	0.943	0.935	0.939	0.978	0.825	0.939	0.953	0.946	0.980	0.678

**Table 3:** Comparison of evaluation metrics for each length estimation method: mean average precision error (MAPE), mean average error (MAE), and root mean square error (RMSE).

Methods	MAPE (%)	MAE (cm)	RMSE (cm)
Hypotenuse	10.87	1.58	1.66
MBBox	1.56	0.23	0.37
Hough Line	2.95	0.44	0.61

**Table 4:** Shrimp counting results.

Captures	MAPE	MAE	RMSE
Feed3	6.45%	0.82	1.20
Feed4	9.23%	1.08	1.50
Feed5	6.95%	1.05	1.41
Feed6	6.03%	0.93	1.39

## Data Availability

The ShrimpFarming dataset is available for research purposes at: http://www.img.lx.it.pt/ShrimpFarming/.
